# Classification of Citrus Huanglongbing Degree Based on CBAM-MobileNetV2 and Transfer Learning

**DOI:** 10.3390/s23125587

**Published:** 2023-06-14

**Authors:** Shiqing Dou, Lin Wang, Donglin Fan, Linlin Miao, Jichi Yan, Hongchang He

**Affiliations:** 1 College of Geomatics and Geoinformation, Guilin University of Technology, Guilin 541006, China; doushiqing@glut.edu.cn (S.D.); wanglin19980829@163.com (L.W.); 13253524923@163.com (L.M.); hhe@glut.edu.cn (H.H.); 2 Ecological Spatiotemporal Big Data Perception Service Laboratory, Guilin University of Technology, Guilin 541006, China; 3 College of Mechanical and Control Engineering, Guilin University of Technology, Guilin 541006, China; 18646104988@163.com

**Keywords:** citrus huanglongbing, MobileNetV2, CBAM, transfer learning, disease classification, image recognition

## Abstract

Citrus has become a pivotal industry for the rapid development of agriculture and increasing farmers’ incomes in the main production areas of southern China. Knowing how to diagnose and control citrus huanglongbing has always been a challenge for fruit farmers. To promptly recognize the diagnosis of citrus huanglongbing, a new classification model of citrus huanglongbing was established based on MobileNetV2 with a convolutional block attention module (CBAM-MobileNetV2) and transfer learning. First, the convolution features were extracted using convolution modules to capture high-level object-based information. Second, an attention module was utilized to capture interesting semantic information. Third, the convolution module and attention module were combined to fuse these two types of information. Last, a new fully connected layer and a softmax layer were established. The collected 751 citrus huanglongbing images, with sizes of 3648 × 2736, were divided into early, middle, and late leaf images with different disease degrees, and were enhanced to 6008 leaf images with sizes of 512 × 512, including 2360 early citrus huanglongbing images, 2024 middle citrus huanglongbing images, and 1624 late citrus huanglongbing images. In total, 80% and 20% of the collected citrus huanglongbing images were assigned to the training set and the test set, respectively. The effects of different transfer learning methods, different model training effects, and initial learning rates on model performance were analyzed. The results show that with the same model and initial learning rate, the transfer learning method of parameter fine tuning was obviously better than the transfer learning method of parameter freezing, and that the recognition accuracy of the test set improved by 1.02~13.6%. The recognition accuracy of the citrus huanglongbing image recognition model based on CBAM-MobileNetV2 and transfer learning was 98.75% at an initial learning rate of 0.001, and the loss value was 0.0748. The accuracy rates of the MobileNetV2, Xception, and InceptionV3 network models were 98.14%, 96.96%, and 97.55%, respectively, and the effect was not as significant as that of CBAM-MobileNetV2. Therefore, based on CBAM-MobileNetV2 and transfer learning, an image recognition model of citrus huanglongbing images with high recognition accuracy could be constructed.

## 1. Introduction

Citrus is the largest fruit tree species in the world and has become a pivotal industry for the efficient development of agriculture and increasing farmers’ incomes in the main producing areas in southern China [1]. Citrus huanglongbing is a devastating disease that endangers the healthy development of the citrus industry; it can infect all citrus species and is currently incurable. Citrus huanglongbing has become a challenge for fruit farmers [2,3,4]. The rapid and accurate identification of citrus huanglongbing is important for improving citrus quality and industrial benefits. The daily management of precision agriculture in the future is bound to be inseparable from mobile devices such as mobile phones, and lightweight network models in deep learning have a broad market space.

As one of the most effective methods in deep learning (DL), the convolutional neural network (CNN) has become a research hotspot in many scientific fields, including agriculture [5,6]. Many scholars have used classic CNN models for crop disease image recognition and achieved better results than traditional recognition methods. Jeon et al. [7] employed the GoogleNet model to automatically extract features to classify and identify plant leaves and obtain good recognition accuracy. Gulzar et al. [8] used the pre-trained VGG16 convolutiaonal neural network to classify and identify 14 known seeds, and the classification accuracy reached 99%. Brahimi et al. [9] collected approximately 15,000 images of tomato leaf diseases and divided them into nine types of diseases based on AlexNet, achieving good recognition results. However, these CNNs can exhibit superior performance only when the network structure is complex and the number of training samples is sufficient. When the number of training samples is small, it becomes prone to overfitting and falling into a local optimal solution. The addition of transfer learning has solved various problems caused by a lack of training samples and has been widely utilized in the field of image recognition. Sun et al. [10], Zhang et al. [11], Long et al. [12], Feng et al. [13], Li [14], Su et al. [15], Zheng et al. [16], and Li et al. [17] achieved good results in the disease identification of Camellia oleifera, cotton, wheat, apples, corns, and grapes by building models based on CNNs and transfer learning under the condition of using small datasets, and indicated that CNNs combined with transfer learning can overcome the difficulty of obtaining large samples and can save considerable amounts of time [18]. The traditional convolutional neural network has many parameters and high complexity, so it is not suitable for plant disease identification applications on mobile devices (such as mobile phones), and mobile devices for crop disease identification are more consistent with people’s actual needs. Therefore, building lightweight models with fewer parameters, strong generalization ability, high recognition accuracy, and smaller sizes is the trend of crop leaf disease identification on mobile terminals. MobileNetV2 [19] is a lightweight network model launched by Google that aims to greatly reduce the size of the model and accelerate the calculation speed of the model without sacrificing model performance. Compared with other networks such as MobileNetV1, InceptionV3, and DenseNet121, MobileNetV2 not only has a higher accuracy rate but also a smaller model that can be deployed in devices with low power consumption and limited computing (such as mobile phones). da Silva et al. [20] used the MobileNetV2 model to classify and identify three types of citrus diseases and one type of healthy citrus, and compared MobileNetV2 with EfficientNetV2-B0 and NASNet-Mobile to conclude that MobileNetV2 had a good balance between accuracy and timing to perform this task in a mobile application. Liu et al. [21] designed and implemented a mobile-based corn disease recognition system, which identified corn diseases based on transfer learning and the MobileNetV2 model. The average recognition accuracy of corn leaf diseases was 84%, and the time spent was only 1.16 s. Gulzar [22] developed a deep learning model, TL-MobileNetV2, based on MobileNetV2 architecture. In the TL-MobileNetV2 model, five different layers were added after removing the classification layer that was present in the MobileNetV2 architecture to improve the efficiency and accuracy of the model. A dataset consisting of forty types of fruits was used to train and test the proposed model. The experimental results showed that the TL-MobileNetV2 performed well on the fruit dataset by attaining 99% accuracy. Zhang et al. [23] selected the MobileNetV2 model to judge the maturity of grapes and obtained a classification accuracy of 87%, which was conducive to promoting the automation of grape picking. Yang et al. [24] identified corn disease images based on transfer learning and the MobileNetV2 model, and (via experimental comparison) determined that the transferred MobileNetV2 model had the best recognition effect on small corn disease samples and could reduce the computational load of the model and greatly shorten the recognition time. Xiang et al. [25] utilized a lightweight convolutional neural network, MobileNetV2, based on transfer learning technology to classify and recognize fruit images, and obtained a classification accuracy rate of 85.12%. The application scenarios of the abovementioned experiments based on MobileNetV2 are mostly limited to simple laboratory environments. For this reason, some scholars have carried out research on leaf disease identification in the field environment to address this problem. Chen et al. [26] divided the diseases into easy-to-see diseases and subtle diseases, according to the size of the disease spots, and further enhanced the features of subtle diseases on the leaf surface. Based on the improved MobileNetV2, multiple types of crop diseases were identified, and good identification was obtained. Hossain et al. [27] redesigned the DSCPLD model that is suitable for plant leaf diseases based on the depth-wise separable convolution module in MobileNetV2 and achieved good recognition results in leaf images with complex backgrounds. Liu et al. [28] embedded the SK module behind the backbone of MobileNetV2, which improved the ability to learn leaf lesions of target plants with different leaf areas. Although the above research overcame the limitation of the simple background environment and reduced the number of model parameters, MobileNetV2 only relied on high-level information in the network for feature analysis when identifying diseases in the complex background environment and paid less attention to local information and spatial information. Additionally, there was the problem of scattered areas of interest. The CBAM is a lightweight and general module that can be seamlessly integrated into any CNN architecture with negligible overhead and is end-to-end trainable with base CNNs, where the channel attention module decides what to focus on and the spatial attention module decides where to focus. The combination of the CBAM and MobileNetV2 can effectively improve the network’s attention to the local information and spatial information of images, and further improve the feature extraction ability of the network.

This paper establishes a sample set based on the early, middle, and late stages of citrus huanglongbing leaf images collected in multiple orchards in Yongfu County, Guilin City, Guangxi District, in December 2021 and proposes an improved lightweight deep learning model, CBAM-MobileNetV2, to realize the early identification and classification of citrus huanglongbing. First, the MobileNetV2 convolutional features were extracted to capture the high-level object-based information and an attention module was employed to capture the interesting semantic information. Second, the convolution and attention modules were combined to fuse these two types of information. Last, a new fully connected layer and a softmax classification layer were established, and the global average pooling layer, batch normalization layer, and dropout layer were added to the newly established fully connected layer to improve the recognition accuracy while improving the model training speed. To verify the performance of the model, CBAM-MobileNetV2 was simultaneously compared with three network models (MobileNetV2, InceptionV3 [29], and Xception [30]), and two different transfer learning methods and three different initial learning rates were selected to form 24 groups of experimental programs for training. The best model was selected. This approach provided model building technical support for the development of an intelligent recognition system for citrus huanglongbing based on mobile terminals.

The main contributions of this paper are as follows:

- We created the citrus huanglongbing image dataset ourselves, and based on an improved lightweight deep learning model, we could classify the early, middle, and late stages of citrus huanglongbing leaf images with different degrees of the disease, so as to realize the early identification and classification of citrus huanglongbing.
- We proposed a lightweight deep learning model, CBAM-MobileNetV2, which combined the pretrained MobileNetV2 and an attention module. The convolutional module captured object-based high-level information, while the attention module paid more attention to specific salient regions compared to the convolutional module. Therefore, the convolutional module and attention module delivered complementary information which can more effectively classify the disease severity of citrus huanglongbing.
- Our proposed method requires a smaller number of trainable parameters as we leverage the pre-trained weights for all layers of the MobileNetv2 architecture. This makes our model more suitable for deployment on resource-constrained devices.
- We compared MobileNetV2, InceptionV3, and Xception with the model proposed in this paper. The experimental results showed that CBAM-MobileNetV2 had stable performance on the citrus huanglongbing dataset and outperformed other lightweight models. Furthermore, dropout and data augmentation techniques were incorporated to minimize the chances of overfitting.

The rest of this article is organized as follows. In Section 2, a description of the dataset is reported and discussed. In Section 3, the model selection, proposed model, transfer learning, and evaluation metrics are reported and discussed. In Section 4, model tuning, experimental settings, and experimental results and analysis are provided, whereas Section 5 provides the conclusion.

## 2. Materials

### 2.1. Image Acquisition and Selection

The image data of citrus leaves used in this study were collected by Shiqing Dou and Lin Wang in December 2021 in multiple orchards in Yongfu County, Guilin City, Guangxi Zhuang Autonomous Region. The citrus varieties collected include Fertile orange, Sugar orange, and so on. According to the onset characteristics of citrus huanglongbing, leaf images of citrus huanglongbing to different degrees in the early, middle, and late stages were collected. The images were taken with a Huawei P30 mobile phone, a device manufactured by Huawei Technologies Co., Ltd. in Shenzhen, China. The size of the images was 3648 pixels × 2736 pixels. As the imbalance of sample categories had a great impact on the performance of the CNNs, the number of leaves of each degree of citrus huanglongbing was basically balanced. After 3 shots, 751 clear images were selected as the original images and were divided into 3 types of labels, based on the different degrees of disease in the early, middle, and late stages.

### 2.2. Image Augmentation

To improve the robustness and generalization ability of model training, this study employed the ImageDataGenerator class provided in Keras to perform data augmentation on the collected original image dataset of citrus huanglongbing. Augmentation methods included random brightness increases, angular rotations, translational transformations, flipping, and scaling. A total of 751 original images were enhanced and expanded to 6008 images, and the image size was uniformly adjusted to 512 × 512; 80% of all images were combined into a training set and 20% were combined into a test set. Table 1 shows the specific distribution of the data in this dataset.

## 3. Construction of the Classification Model for Citrus Huanglongbing Disease Severity

### 3.1. The Convolution Module

**Figure 1:**
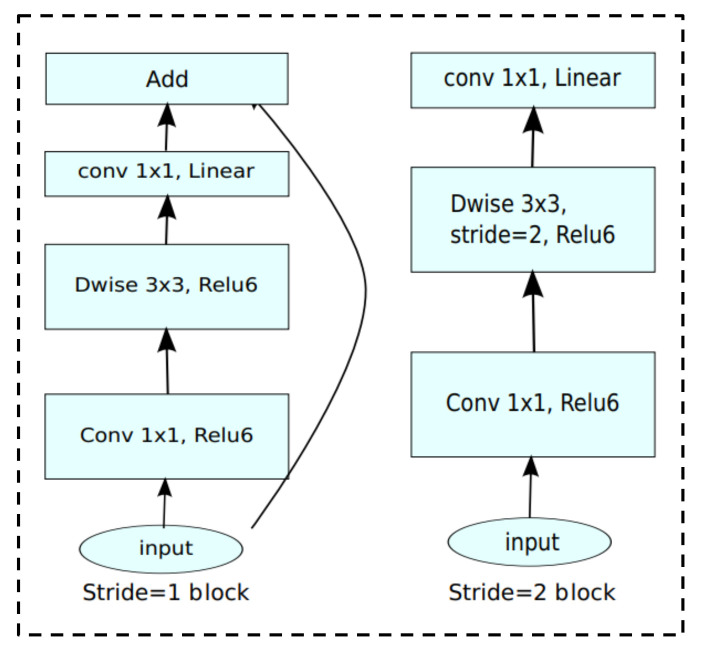
Inverted residuals and linear bottlenecks.

A CNN is a feedforward neural network. Its basic structure consists of an input layer, a convolutional layer, a pooling layer, a fully connected layer, and an output layer. Convolution and pooling are the core operations. The function of a convolution layer is mainly to extract local features of the input image, and the functions of a pooling layer are mainly to reduce the feature dimension, compress the number of data and parameters, reduce overfitting, and improve the fault tolerance of the model. The use of CNNs for image recognition and classification has not only become a trend in the field of agriculture, but also in other fields such as scene image recognition [31], remote sensing image analysis [32], and so on. Therefore, convolution operations are major contributors to computer vision tasks, but when the network structure is deeper and larger, such as VGG-16, VGG-19, Alexnet, and Xception, the computational cost of convolution operation is high. The MobileNetV2 network is a lightweight CNN focused on mobile or embedded devices, which was proposed by the Google team in 2018. Its structural parameters are shown in Table 2. The MobileNetV2 network has 17 bottleneck layers, the core of which is depth-wise separable convolution (DSC), including depth-wise convolution and point-by-point convolution. Compared with MobileNetV1, the MobileNetV2 network model introduces inverted residuals and linear bottlenecks. The linear bottleneck module effectively prevents the ReLu function from destroying information in low-dimensional space and directly outputs the information after passing through the point-by-point convolution layer. The inverse residual structure is the opposite of the ResNet structure. First, a 1 × 1 convolution is used to achieve dimensionality increase; second, a 3 × 3 DW convolution is used for feature extraction; and last, a 1 × 1 convolution is used to achieve dimensionality reduction, ensuring the feature extraction in high-dimensional space. There is a shortcut connection only when stride = 1 and the input feature matrix is the same shape as the output feature matrix, as shown in Figure 1. Compared with traditional CNNs, the MobileNetV2 network can greatly reduce the model parameters and the amount of calculation under the premise of a small decrease in accuracy.

### 3.2. Convolutional Block Attention Module

The attention mechanism allows the network to learn to pay attention to the key information in images and can suppress other useless information, which greatly improves the efficiency of image classification. The convolutional block attention module (CBAM) is a lightweight attention module [33] that consists of a channel attention module (CAM) and a spatial attention module (SAM).

#### 3.2.1. Channel Attention Module

**Figure 2:**
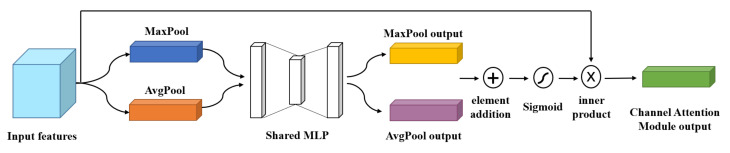
Channel attention module.

First, the channel attention module compresses the input features in the spatial dimension and performs global max pooling and global average pooling based on width and height, respectively. The two pooled one-dimensional vectors are inputted into the shared multilayer perceptron (shared MLP) model, and the corresponding elements of the MLP output features are summed one by one. Second, through the sigmoid activation function, inner product operation is performed with the initial feature map. The output feature map is the input feature required by the spatial attention module, as shown in Figure 2.

#### 3.2.2. Spatial Attention Module

**Figure 3:**
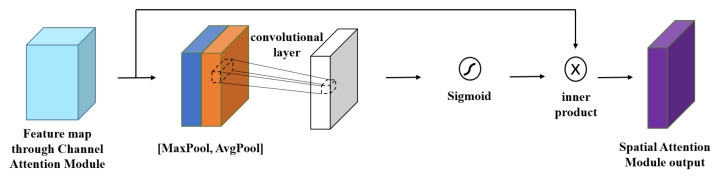
Spatial attention module.

The feature map outputted by the channel attention module serves as the input feature map of this module. First, global max pooling and global average pooling are performed based on the channel, and the two obtained feature maps are merged in the channel-based dimension. Second, a 7 × 7 convolution operation is performed. After the sigmoid activation function, the inner product operation is performed on the output feature map and the feature map inputted by the spatial attention module to obtain the final generated features, as shown in Figure 3.

### 3.3. ImageNet Dataset

The ImageNet dataset is a large-scale, computer vision dataset that was established to promote the development of computer image recognition technology. The ImageNet dataset, which has always been a benchmark for evaluating the performance of image classification algorithms, was created under the leadership of Professor Li Feifei from Stanford University. The pictures in this dataset cover most of the picture categories that you will see throughout life, including 14,197,122 pictures and 21,841 Synset indices, and each picture is manually labelled.

### 3.4. Transfer Learning

Transfer learning (TL) is a machine learning method that involves a trained convolutional neural network model being transferred to another domain (target domain) so that the target domain can achieve better learning results. Using transfer learning, the general parameters of the ImageNet model obtained from numerous experiments were transferred to the experiment to realize the classification and recognition of the disease degree of citrus huanglongbing leaves. The experiment compares the transfer learning method of parameter fine tuning and parameter freezing and selects the model transfer method with the best effect.

#### 3.4.1. Parameter Fine Tuning

Parameter fine tuning redesigns the convolution module of the model so that it can be transferred, freezes some layers of the network, and retrains some layers. In layman’s terms, the initialization network of the pretrained network is used to train part of the network or the whole network with new data. The experiment freezes all the layers before the 10th bottleneck of the MobileNetV2 network and then restrains the subsequent bottleneck. As the underlying network captures common features such as curves and edges, the information extracted at the bottom of the general convolutional neural network is the same. These weights are kept unchanged, the network focusses on some unique features of the citrus huanglongbing dataset during the learning process, and the network is adjusted so it can be trained later until the fine-tuning method with the highest accuracy is identified as the final fine-tuning model.

#### 3.4.2. Parameter Freezing

Parameter freezing involves freezing the weights of all networks in the target domain, with the exception of the fully connected layers, thus replacing the fully connected layers of the model in the source domain with new fully connected layers with random weights, and only training the new fully connected layers. In this experiment, the MobileNetV2 network that was trained on ImageNet served as the feature extractor of the experimental task, and only the last redesigned fully connected module was learned, while the pretrained network parameters were not modified or frozen.

### 3.5. Model Building and Improvement

**Figure 4:**
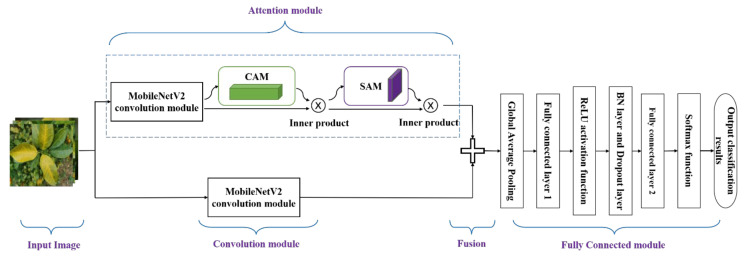
Flow chart of the recognition model for citrus huanglongbing degree classification based on CBAM-MobileNetV2.

Since the images of citrus huanglongbing leaves employed in this experiment had complex background characteristics and different sizes of lesion areas, MobileNetV2 had a shortage of scattered regions of interest with this type of dataset. Therefore, this experiment aimed to overcome the shortcoming of MobileNetV2 in identifying leaf diseases in complex backgrounds and improving the MobileNetV2 network. As shown in Figure 4, the improved model was named CBAM-MobileNetV2, and was mainly divided into five parts: the input image, the convolution module, the attention module, fusion, and the fully connected module. In the convolution module, the MobileNetV2 network pretrained on the ImageNet dataset was utilized as the backbone network, and all layers of the network were frozen to the last convolution layer before the fully connected layer, and a feature map of size 7 × 7 × 1280 was generated. In the attention module, the feature map generated by the convolution module served as the input feature of the CBAM, and a feature map with a size of 7 × 7 × 1280 was obtained after training the attention module. The feature maps obtained from the convolutional module and the attention module were then fused to obtain a combined feature map. In the fully connected module, the newly designed fully connected layer was employed to replace the fully connected layer module of MobileNetV2, and the global average pooling layer, the batch normalization layer, and the dropout layer were added to the new classifier to improve the recognition accuracy while improving the model training speed. The new model was trained with the preprocessed citrus huanglongbing leaf image dataset, and the trained new model could classify and recognize citrus huanglongbing leaf images of different degrees.

To verify the performance of the model, on the basis of improving and constructing CBAM-MobileNetV2, this study also selected and compared three models: MobileNetV2, InceptionV3, and Xception. The fully connected layers of these three models adopted the same improvement method as the CBAM-MobileNetV2 model, and the rest were not improved. The InceptionV3 model is also a lightweight network, and the Xception model evolved from InceptionV3. These models can be compared against the improved MobileNetV2 model. The InceptionV3 model consists of 11 Inception modules with 47 layers. The Xception model is divided into 3 flows, namely the entry flow, the middle flow, and the exit flow. The model is also divided into 14 blocks: 4 blocks in the entry flow, 8 blocks in the middle flow, and 2 blocks in the exit flow. All three models also use transfer learning, remove the original classifier, and then replace it with a new classifier for different degrees of citrus huanglongbing leaf image recognition tasks. In the transfer learning method of parameter fine tuning, the MobileNetV2 network model is fine-tuned in the same way as the CBAM-MobileNetV2 network. In this experiment, all the layers before the ninth Inception module of the InceptionV3 network were frozen, the subsequent Inception modules were retrained, and the network to be trained was adjusted until the fine-tuning method with the highest accuracy was identified as the final fine-tuning model of InceptionV3. In this experiment, all the layers before the 11th block of the Xception network were frozen, the following blocks were retrained, and the network to be trained was adjusted until the fine-tuning method with the highest accuracy was identified as the final fine-tuning model of Xception.

### 3.6. Evaluation Metrics

To verify the quality of the model, this paper applied the average accuracy evaluation index of model recognition to evaluate the performance of different methods on the dataset. The formula for calculating the average accuracy of model recognition is presented as follows:

(1) $$\mathrm{accuracy}=\frac{1}{n}\sum_{i=1}^{n}\frac{N_{ci}}{N_{Ti}},$$

In the formula, $N_{ci}$ represents the number of correct predictions of class *i*, $N_{Ti}$ represents the total number of samples of class *i*, and n is the total number of sample categories.

## 4. Model Training and Analysis of Experimental Results

### 4.1. Experimental Program

The computer CPU used in this experiment was the Intel(R) Core (TM) i7-9700, with a memory of 16.0 GB, a NVIDIA GeForce GTX1660 GPU, a main frequency of 3.00 GHz, a video memory of 3G, and a Windows 10 operating system. The framework used for modelling and model training in this experiment was TensorFlow2.3.0, and the chosen programming language was Python.

Considering the performance of the hardware device and the training effect, each batch of training and testing in the experiment consisted of 32 images, and the batch size was set to 32. The Adam optimizer was used to optimize the model as it has the advantages of fast convergence speed and easy parameter adjustment. The loss function was SparseCategoricalCrossentrop. In this paper, the network model was trained and analyzed with different influencing factors. First, the impact of two different transfer learning training methods on model performance was analyzed. In the transfer learning method of parameter freezing, 100 epochs were set. In the transfer learning method of parameter fine tuning, all layers of the convolutional base were frozen, only the added classification layer was trained, and 50 epochs were set. Then, a part of the convolutional base layer was unfrozen, the unfrozen convolutional layer and the added classification layer were jointly trained, the learning rate was reduced to 1/10 of the original, and another 50 epochs were set. If fine tuning was performed without a well-trained classifier, the initial training error was expected to be very large and the representations learned by these convolutional layers before fine tuning were destroyed. Second, the recognition performance of different models was analyzed, and the optimal model was screened. Lastly, the impact of the initial learning rate (0.001, 0.0001, and 0.00001) on the performance of the model was analyzed. In summary, there were 24 groups of experiments in total. Table 3 shows the average recognition accuracy of the 24 groups of models after training.

### 4.2. Analysis of Experimental Results

#### 4.2.1. Impact of Transfer Learning Methods on Model Performance

**Figure 5:**
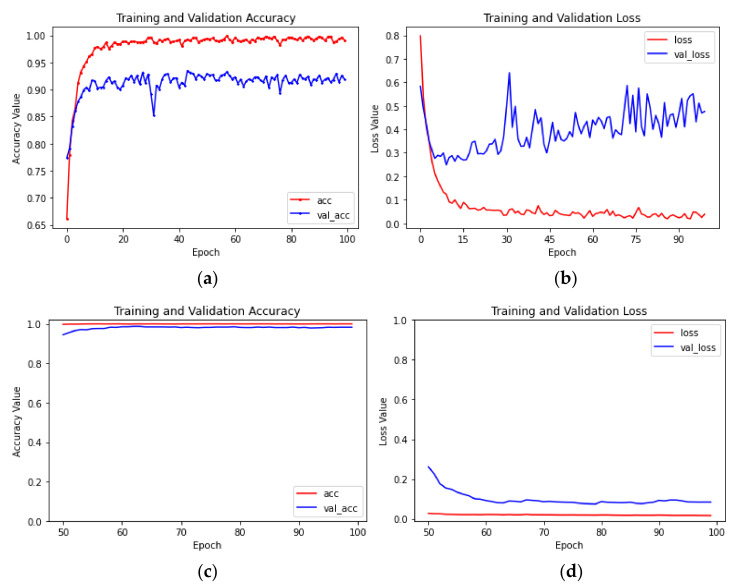
The identification accuracy and loss value curves for Schemes 1 and 13: (**a**) the recognition accuracy of Scheme 1; (**b**) the loss value for Scheme 1; (**c**) the recognition accuracy of Scheme 13; (**d**) the loss value for Scheme 13.

As shown in Table 3, among the 24 groups of models, with the same models and initial learning rates, the transfer learning method of parameter fine tuning was significantly better than the transfer learning method of parameter freezing, and the recognition accuracy of the test set increased by 1.02 percentage points to 13.6 percentage points. With the transfer learning method of parameter fine tuning, the average recognition accuracy rate of the test set of Scheme 13 was the highest, reaching 98.75%. With the transfer learning method of parameter freezing, the average recognition accuracy rate of the test set of Scheme 1 was the highest, which was 93.50%. The accuracy rate and loss value curves of the model training process of Schemes 13 and 1 are shown in Figure 5. In Scheme 1, after 30 iterations, the average recognition accuracy rate of the training set exceeded 99% and then remained stable, while the average recognition accuracy rate of the test set slightly fluctuated, and the loss value curve of the test set exhibited obvious overfitting. The reason for this phenomenon was that in the transfer learning method of parameter freezing, the network structure was too simple, and the model repeatedly entered the local optimal point during training. In Scheme 13, first, all layers of the convolutional base were frozen, and only the added classification layer was trained for 50 epochs. The average recognition accuracy of the training set exceeded 99% after 20 iterations. Second, a part of the convolutional base layer was unfrozen, the learning rate was reduced to 1/10 of the original, and the unfrozen convolutional layer and added classification layer were jointly trained for another 50 epochs. The average recognition accuracy rate of the training set exceeded 99% after one iteration, the average recognition accuracy rate curves of the test set and training set were basically consistent, and a good recognition effect was obtained. The above research showed that in image recognition based on CBAM-MobileNetV2 for classifying the degree of citrus huanglongbing, it was more appropriate to adopt the transfer learning method of parameter fine tuning.

#### 4.2.2. Recognition Performance Analysis of Different Models

**Figure 6:**
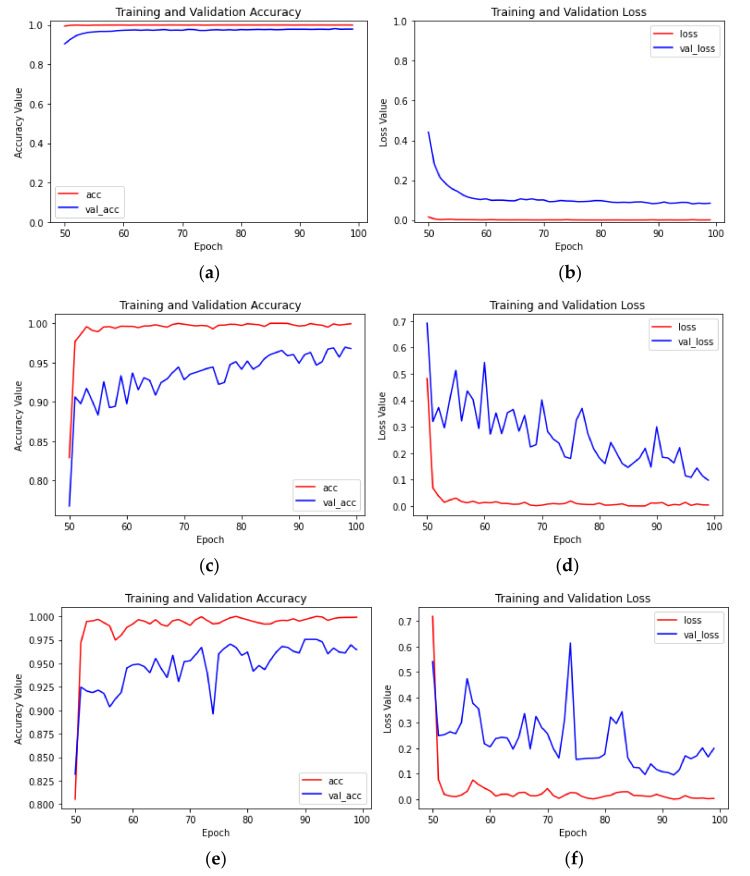
The identification accuracy and loss value curves for Schemes 16, 19 and 22: (**a**) the recognition accuracy of Scheme 16; (**b**) the loss value for Scheme 16; (**c**) the recognition accuracy of Scheme 19; (**d**) the loss value for Scheme 19; (**e**) the recognition accuracy of Scheme 22; (**f**) the loss value for Scheme 22.

As demonstrated by Schemes 13–24 in Table 3, although the fully connected layers of the CBAM-MobileNetV2, MobileNetV2, Xception, and InceptionV3 models showed the same improvement, the average recognition accuracy of the test set trained by the CBAM-MobileNetV2 network model was better, and the time required for training was similar to that of MobileNetV2. The training effect of the four models was the best when the transfer method of parameter fine tuning and an initial learning rate of 0.001 were employed. The average recognition accuracy and loss curves of the CBAM-MobileNetV2 model in Scheme 13 are shown in Figure 5c,d, and the average recognition accuracy curves of the test set and training set were similar. The average recognition accuracy and loss value curves of the MobileNetV2 model in Scheme 16 are shown in Figure 6a,b. The average recognition accuracy curves of the test set and the training set were similar, but the average recognition accuracy of the test set was 98.14%, which differed from the average recognition accuracy of CBAM-MobileNetV2 by 0.61 percentage points. The convergence speed of the loss value curve of MobileNetV2 was lower than that of CBAM-MobileNetV2. The average recognition accuracy and loss value curves of the Xception model of Scheme 19 and the InceptionV3 model of Scheme 22 are shown in Figure 6c–f; the average recognition accuracy rates of the test set were 96.96% and 97.55%, respectively, but the accuracy curve and loss value curve obviously exhibited overfitting. The reason for this phenomenon was that there were inverted residuals in MobileNetV2 which can complete information, reduce information loss, and optimize the direction of gradient descent during training, while the Xception and InceptionV3 models did not have modules similar to the inverted residuals, so there were shocks during the training process. Therefore, this paper chose the CBAM-MobileNetV2 network model to classify leaves of citrus huanglongbing to different degrees.

**Figure 7:**
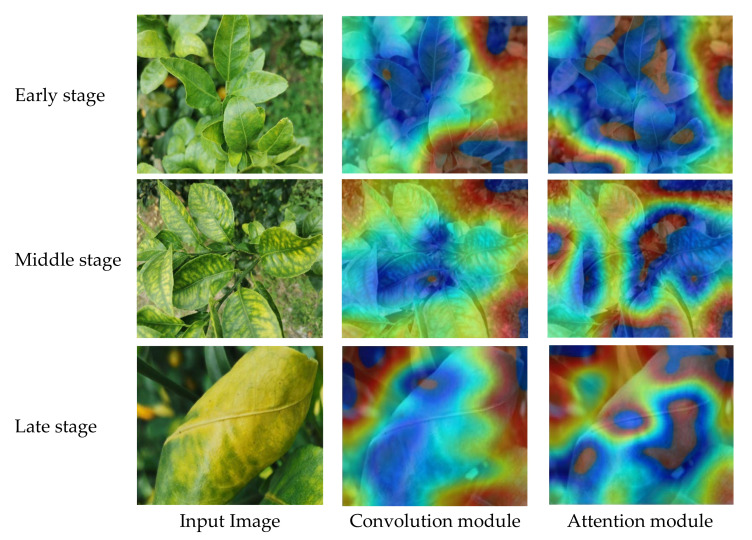
GradCam visualization examples for the early, middle and late stages of Citrus huanglongbing.

In addition, to further compare the ability of the convolution module and attention module to extract disease features, the experiment extracted the class activation map (CAM) of some images after the last convolution layer of the two modules, as shown in Figure 7. The convolution module was rendered at the center of the images, while the attention module focused more on specific salient regions compared to the convolution module. Therefore, the convolutional module and attention module delivered complementary information which can more effectively classify the disease severity of citrus huanglongbing.

**Figure 8:**
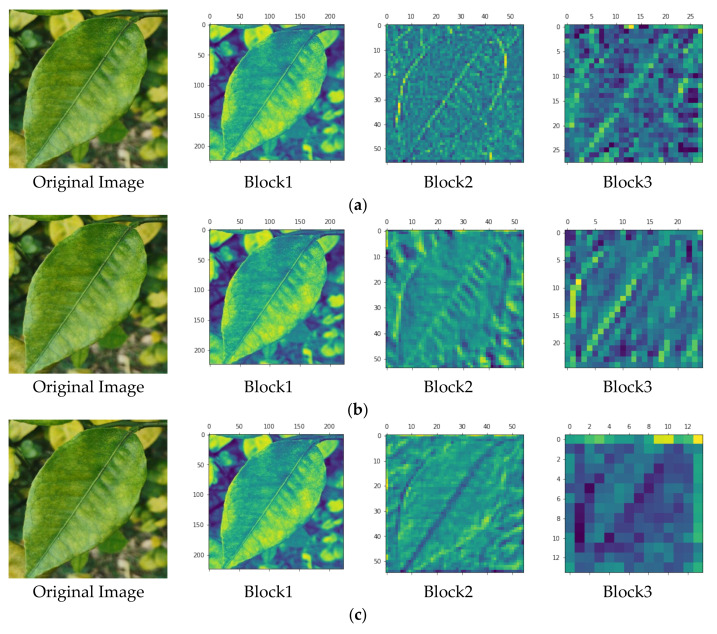
The visualization of feature extraction for convolutional modules with three network structures: (**a**) features extracted by the MobileNetV2 convolutional module; (**b**) features extracted by the InceptionV3 convolutional module; (**c**) features extracted by the Xception convolutional module.

The feature extraction process of the MobileNetV2, InceptionV3, and Xception convolution modules is shown in Figure 8. Block 1, Block 2, and Block 3 in Figure 8 are the feature maps outputted by the image in the three network structures after passing through the front, middle, and back module structures. There was a small difference in the features extracted by the three network structures in the shallow layer, but MobileNetV2 had a stronger ability to extract deep abstract features than the other two models.

#### 4.2.3. Impact of Initial Learning Rates on Model Performance

**Figure 9:**
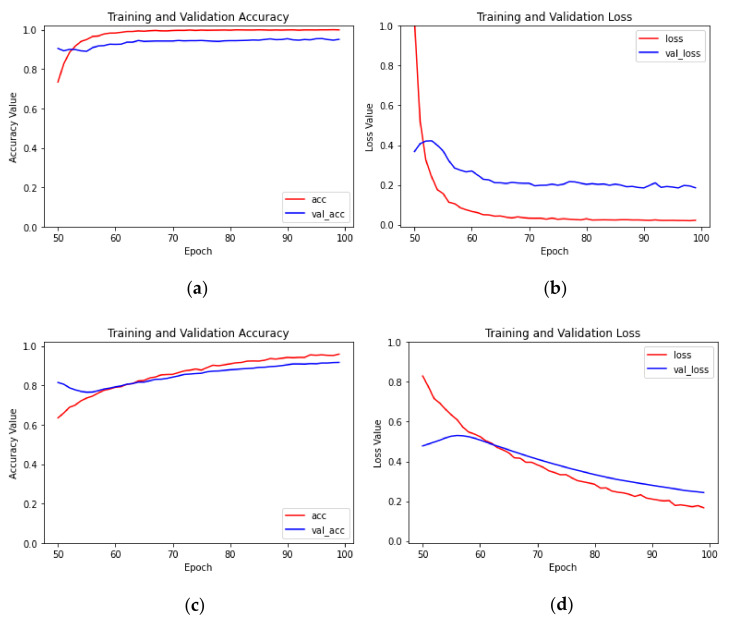
The identification accuracy and loss value curves for Schemes 14 and 15: (**a**) the recognition accuracy of Scheme 14; (**b**) the loss value for Scheme 14; (**c**) the recognition accuracy of Scheme 15; (**d**) the loss value for Scheme 15.

As shown by Schemes 13–15 in Table 3, when the transfer learning method of parameter fine tuning and the CBAM-MobileNetV2 network model were utilized for training, only the average recognition accuracy of the test set of Scheme 13 exceeded 98%. According to the recognition accuracy curves of the model training process with different initial learning rates (Figure 5c and Figure 9), the average recognition accuracy rates of the training set and test set of Scheme 13 with an initial learning rate of 0.001 remained at a relatively high level, and the trend was similar. In Scheme 14, with an initial learning rate of 0.0001, although the average recognition accuracy of the training set and test set were similar, the average recognition accuracy of the test set was very different from that of Scheme 13, and the convergence speed was slower. Scheme 15, with an initial learning rate of 0.00001, had the slowest convergence speed and had not converged after training for 100 epochs. Therefore, the model with an initial learning rate of 0.001 was the best.

#### 4.2.4. Comparison of the Latest Classification Methods for Citrus Diseases

As shown in Table 4, the proposed model, CBAM-MobileNetV2, was compared with the latest classification methods for citrus diseases on both qualitative and quantitative features. In the field of machine learning, Deng et al. [34] used C-SVC to judge whether citrus leaves were infected with huanglongbing. The experimental results showed that the proposed huanglongbing recognition method achieved about 91.93% with low-cost and low-computation complexity. Sharif et al. [35] used M-SVM to classify six citrus diseases obtained from the Citrus disease image gallery dataset, and the recognition accuracy rate was 97.00%. In the field of deep learning, Pan et al. [36] created image datasets of six types of citrus diseases by themselves with the help of experts, and proposed the Simplify DenseNet201 model with a recognition accuracy of 88.77%. Xing et al. [37] built the Weakly DenseNet-16 model to classify 17 types of citrus pests obtained from the Internet and 7 types of citrus diseases created by themselves, and the recognition accuracy rate was 93.33%. Tie et al. [38] constructed the F-ResNet model to classify four types of citrus diseases and one type of citrus healthy leaves, and the recognition accuracy rate was 93.60%. From the table, it can be concluded that our proposed model performed well in citrus disease classification. Based on classification accuracy, our method outperformed the second best performing method (M-SVM [35]) by 1.75% and the worst performing method (Simplify DenseNet201 [36]) by 9.98%.

### 4.3. Ablative Study of the Proposed Method

Table 5 shows the effect of adding the attention module in CBAM-MobileNetV2 under the condition of using the transfer learning method of parameter fine tuning and an initial learning rate of 0.001. From Table 5, it can be seen that both attention and convolution modules were responsible for improving the performance. The average recognition accuracy of the test set was 98.14% if the convolution module only was used, whereas it was 98.75% if the attention module was included. Therefore, the increase in the recognition accuracy of the test set may be attributed to the synergistic effect of attention and convolution modules.

## 5. Conclusions

This paper proposed a lightweight deep learning model CBAM-MobileNetV2, which combined the pretrained MobileNetV2 and an attention module to enable the convenient identification of citrus huanglongbing. The object-based high-level information obtained by the convolution module was fused with the semantic information of interest captured by the attention module to obtain better recognition results. Three network models (MobileNetV2, InceptionV3, and Xception) were selected and compared with CBAM-MobileNetV2, and 24 sets of schemes were set up for model training. By analyzing the impact of different transfer learning methods, different model training effects and initial learning rates on model performance, the model with high recognition accuracy was selected. According to the experimental results, the following conclusions were obtained:

(1) Based on CBAM-MobileNetV2 and transfer learning, the recognition accuracy of the citrus huanglongbing leaf image recognition model of different degrees reached 98.75%, and a very good recognition effect was achieved. The effect was significantly better than that of the MobileNetV2, Xception, and InceptionV3 network models. The convolutional module of CBAM-MobileNetV2 captured object-based high-level information, while the attention module paid more attention to specific salient regions compared to the convolutional module. Therefore, the convolutional module and attention module delivered complementary information which can more effectively classify the disease severity of citrus huanglongbing.
(2) With the same model and initial learning rate, the transfer learning method of parameter fine tuning was significantly better than the method of parameter freezing, and the recognition accuracy of the test set increased by 1.02 to 13.6 percentage points, which showed that the transfer learning method of parameter fine tuning was more suitable for recognizing citrus huanglongbing.
(3) The learning rate was found to have a great impact on the convergence and recognition accuracy of the model. In the transfer learning method of parameter fine tuning, when the learning rate was 0.001, the effect was the best. Therefore, choosing an appropriate learning rate is very important for training the model. In addition, when the collected image samples are preprocessed and data augmentation is completed, the difference in the field shooting scale and shooting angle should be taken into account, and the model performance can be improved by appropriately increasing the sample size.

Our method has some limitations. First, in this experiment, our method only used traditional data augmentation. Second, we only developed a handheld crop pest and disease detector and applied for a patent, but we did not develop an application for detecting diseases. In future work, first, we plan to use the generative adversarial network (GAN) for data augmentation to further improve the model performance. Second, we plan to develop an application for detecting various diseases and combine this application with the handheld crop pest and disease detector for the agricultural field. This mobile-based application will help people with limited knowledge to classify and identify degrees of disease of different types of fruits, so as to promptly diagnose and effectively control various diseases. Third, we plan to use the network model of the yolo series to estimate the yield of citrus fruit trees (including target detection tasks and semantic segmentation tasks), so as to efficiently and accurately understand the estimated annual output of citrus fruit.

## Figures and Tables

**Table 1:** Details of the citrus huanglongbing image dataset.

Degrees of Disease	Number of Original Images	Number of Images after Augmentation	Number of Images	
			Training Set	Test Set
Early stage	295	2360	1888	472
Middle stage	253	2024	1619	405
Late stage	203	1624	1299	325

**Table 2:** The network structure parameters of MobileNetV2.

Number of Layers	Input Size	Operator and Convolution Kernel	N	S
1	224 × 224 × 3	Conv2d 3 × 3	1	2
2	112 × 112 × 32	Bottleneck 3 × 3 1 × 1	1	1
3–4	112 × 112 × 16	Bottleneck 3 × 3 1 × 1	2	2
5–7	56 × 56 × 24	Bottleneck 3 × 3 1 × 1	3	2
8–11	28 × 28 × 32	Bottleneck 3 × 3 1 × 1	4	2
12–14	14 × 14 × 64	Bottleneck 3 × 3 1 × 1	3	1
15–17	14 × 14 × 96	Bottleneck 3 × 3 1 × 1	3	2
18	7 × 7 × 160	Bottleneck 3 × 3 1 × 1	1	1
19	7 × 7 × 320	Conv2d 1 × 1	1	1
20	7 × 7 × 1280	Avgpool 7 × 7	1	-
21	1 × 1 × 1280	Conv2d 1 × 1	1	-

N represents the number of repetitions. S represents the stride of the first layer. Conv2d is a convolution operation, Bottleneck is a structure that increases the feature dimension first and then decreases, and Avgpool is a global average pooling operation.

**Table 3:** Recognition accuracy of the model.

Scheme No.	Transfer Learning Method	Model	Initial Learning Rate	Number of Parameters	Training Time	Recognition Accuracy	
						Training Set	Test Set
1	Parameter freezing	CBAM-MobileNetV2	0.001	453,574	419.01 m	99.83	93.50
2			0.0001		425.21 m	100.00	92.31
3			0.00001		423.54 m	95.40	89.70
4		MobileNetV2	0.001	329,219	413.02 m	99.60	93.16
5			0.0001		421.03 m	99.92	92.15
6			0.00001		425.93 m	86.90	83.11
7		Xception	0.001	525,827	765.95 m	98.80	86.74
8			0.0001		772.00 m	99.63	90.03
9			0.00001		765.48 m	78.81	75.59
10		InceptionV3	0.001	525,827	531.60 m	99.12	88.34
11			0.0001		520.22 m	99.52	88.68
12			0.00001		501.13 m	80.37	75.93
13	Parameter Fine tuning	CBAM-MobileNetV2	0.001	2,371,462	480.25 m	100.00	98.75
14			0.0001		476.05 m	100.00	95.52
15			0.00001		473.87 m	95.85	92.48
16		MobileNetV2	0.001	2,191,811	472.38 m	100.00	98.14
17			0.0001		478.96 m	100.00	94.85
18			0.00001		466.62 m	95.31	92.15
19		Xception	0.001	10,004,171	964.67 m	100.00	96.96
20			0.0001		982.75 m	100.00	91.05
21			0.00001		973.13 m	92.98	87.58
22		InceptionV3	0.001	14,149,699	633.37 m	100.00	97.55
23			0.0001		596.86 m	100.00	91.30
24			0.00001		603.23 m	99.75	89.53

**Table 4:** Comparison with the latest classification methods for citrus diseases.

Classification Method	Dataset	Classes	Availability	Accuracy
C-SVC [34]	Self-created [34]	2	Private	91.93%
M-SVM [35]	Citrus disease image gallery [35]	6	Public	97.00%
Simplify DenseNet201 [36]	Self-created [36]	6	Private	88.77%
Weakly DenseNet-16 [37]	Self-created [37]	24	Public	93.33%
F-ResNet [38]	PlantVillage and self-created [38]	5	Private	93.60%
CBAM-MobileNetV2	Self-created	3	Private	98.75%

**Table 5:** Ablative study of our proposed model.

Transfer Learning Method	Initial Learning Rate	Attention (with or without)	Recognition Accuracy	
			Training Set	Test Set
Parameter fine tuning	0.001	Attention (with)	100.00	98.75
		Attention (without)	100.00	98.14

## Data Availability

The data are not publicly available due to privacy restrictions.

## Abbreviations

The following abbreviations are used in this manuscript:

CAM	channel attention mechanism
CBAM	convolutional block attention module
CNN	convolutional neural network
CPU	central process unit
DL	deep learning
DSC	depth-wise separable convolution
GAN	generative adversarial network
GPU	graphics processing unit
MLP	multilayer perceptron
SAM	spatial attention mechanism
TL	transfer learning
